# The burden of informal caregiving in Hungary, Poland and Slovenia: results from national representative surveys

**DOI:** 10.1007/s10198-019-01058-x

**Published:** 2019-05-14

**Authors:** Petra Baji, Dominik Golicki, Valentina Prevolnik-Rupel, Werner B. F. Brouwer, Zsombor Zrubka, László Gulácsi, Márta Péntek

**Affiliations:** 1 Department of Health Economics, Corvinus University of Budapest, Fővám tér 8, 1093 Budapest, Hungary; 2 Department of Experimental and Clinical Pharmacology, Medical University of Warsaw, ul. Banacha 1b, 02-097 Warsaw, Poland; 3 Institute for Economic Research, Kardeljeva ploščad 17, 1000 Ljubljana, Slovenia; 4 Erasmus School of Health Policy and Management (ESHPM), Erasmus University Rotterdam, PO Box 1738, 3000 DR Rotterdam, The Netherlands; 5 Doctoral School of Business and Management, Corvinus University of Budapest, Fővám tér 8, 1093 Budapest, Hungary

**Keywords:** Informal care, Quality of life, CarerQol, EQ-5D-5L, I19

## Abstract

**Background:**

We aimed to investigate the burden of informal care in Hungary (HU), Poland (PL) and Slovenia (SI).

**Methods:**

A cross-sectional online survey was performed involving representative samples of 1000 respondents per country. Caregiving situations were explored; health status of informal caregivers/care recipients and care-related quality of life were assessed using the EQ-5D-5L and CarerQol-7D.

**Results:**

The proportion of caregivers was (HU/PL/SI) 14.9, 15.0 and 9.6%, respectively. Their mean age was 56.1, 45.6 and 48.0, and the average time spent on informal care was 27.6, 35.5 and 28.8 h/week. Chronic care was dominant (> 1 year: 78.5%, 72.0%, 74.0%) and care recipients were mainly (own/in-law) parents. Average EQ-5D-5L scores of care recipients were 0.53, 0.49 and 0.52. For Poland and Slovenia, EQ-5D-5L scores of informal care providers were significantly lower than of other respondents. Average CarerQol-7D scores were (HU/PL/SI) 76.0, 69.6 and 70.9, and CarerQol-VAS was 6.8, 6.4 and 6.6, respectively. Overall, 89, 87, and 84% of caregivers felt some or a lot fulfilment related to caring. Problems with combining tasks with daily activities were most important in Hungary and Slovenia. Women had a higher probability of being a caregiver in Hungary. CarerQol-7D scores were significantly associated with caregivers’ EQ-5D-5L scores. In Hungary and Poland, living in a larger household was positively, while caring for patients with mental health problems was negatively associated with CarerQol-7D scores.

**Conclusions:**

These first results from the Central and Eastern European region using preference-based measures for the evaluation of informal care can serve as a valuable input for health economic analyses.

## Introduction

Most diseases, especially chronic conditions, which limit patients in their daily activities, create burden not only for patients but for their families as well. Informal care is the care provided for a family member or friend who needs support due to an illness, disability or infirmity of old age. It is mostly non-professional and unpaid care. Informal carers typically are relatives, friends or acquaintances of the persons receiving care or support. Informal care involves different types of tasks such as domestic help, personal care or nursing care; emotional support, supervision; accompanying on visits or performing administrative tasks. Caregiving is often time-consuming: the time spent on caregiving can range from a couple of hours to more than 40 h per week. Furthermore, informal carers often provide care for a long period of time, in some cases for many years [1].

Providing informal care can, therefore, affect caregivers’ quality of life (QoL) [2]. On the one hand, it can be rewarding [3, 4]; on the other hand, it may be difficult to carry out caregiving tasks in combination with other daily activities. Providing informal care can lead to mental health problems such as stress, fear, gloominess, depression, and concerns about the future and the caregiving tasks. Caregiving can also affect physical health of the caregiver, as they can be more often sick, tired or can experience physical stress [2]. Furthermore, other family members may also suffer from health losses because someone in his or her social environment is ill, regardless of his or her care-giving status, which is called “family effect” [5].

Studies on the burden of informal caregiving are scarce in the Central–Eastern European (CEE) region. Available studies mostly focus on informal care volume (i.e,. share of people receiving informal care, care time in hours per week) in specific diseases based on self-reports of the patients [6–13]. Less is known about the overall burden of informal caregiving among the general population, especially from the caregivers’ perspective and including QoL effects of caregiving. Moreover, there is a scarcity of preference-based data on caregivers’ and care recipients’ health status and care-related QoL expressing the utility of these states. Such figures are required in the context of economic evaluations of new treatments and health interventions. The Survey of Health, Retirement and Ageing in Europe (SHARE) project collects data on the provision of informal care among the population aged 50 or over. Based on these data, OECD Health at Glance reports that the prevalence of informal caregivers in the population aged 50 and above is 17.7% in the Czech Republic, 16.2% in Hungary, 12.8% in Poland and 14.6% in Slovenia [14]. According to the European Quality of Life Survey (EQLS) from 2016, the proportion of self-reported informal caregivers in the population aged 18 or above providing informal care at least once a week was 9% in Romania and in the Czech Republic, 10% in Bulgaria, 15% in Slovenia, 18% in Hungary, 19% in Slovakia, and 20% in Poland [15].

Estimates of the prevalence of informal caregivers in the general population show a substantial variation across different data sources. For example, OECD Health at Glance 2013 reports that, in Hungary, 16.2% of the 50+ population provided informal care [14], while based on a representative survey of the population, Rubovszky reports that 25.5% of the 18+ population provided informal care in- or outside the household for a relative/friend over 65 [16]. Such differences can partly be explained by different definitions across studies regarding who is considered as a caregiver. This can be restricted to only family members or also include other members of the social network such as friends/neighbours. Restrictions could be used regarding the content of the care provided, the quantity of time spent on providing care, the duration of care, or the care recipient (e.g., only older people or all age groups). Such differences hamper comparisons of study results, also internationally [16].

Against that background, the objectives of this study are to comprehensively explore, measure and compare the burden of informal caregiving in Hungary, Poland and Slovenia, with a special focus on caregivers’ health status and care-related QoL using standard preference-based measures. We aim to investigate the determinants of (1) informal caregiving, (2) time devoted to the provision of informal care and (3) the quality of life of caregivers. For the analyses, we use data from an online three-country survey carried out in Hungary, Poland and Slovenia between November 2018 and January 2019, on representative samples of 1000 respondents per country. To measure the health status and care-related QoL of informal caregivers, we used the EQ-5D-5L and the CarerQoL-7D instruments. Both are validated tools intended for use in economic evaluations [2, 17].

Such data can be used as inputs to health economic analyses to compare different treatment options and interventions from the societal perspective, taking into account the burden and benefits of informal caregiving as well.

## Methods

### Study design and sample

A representative, cross-sectional, internet-based survey was carried out between November 2018 and January 2019 in Hungary (HU), Poland (PL) and Slovenia (SI). Ethical approval was obtained from the Hungarian Medical Research Council (No. 35286-2/2018/EKU), the Bioethical Committee of Medical University of Warsaw in Poland (AKBE/204/2018) and by the National Medical Ethics Committee (No. 0120-458/2018/4) in Slovenia. Recruitment and data collection were carried out by a survey company, Big Data Scientist Kft. The target sample size was 1000 respondents per country. Quotas were applied to ensure the representativeness of the sample by age, gender, age, educational level, and residency.

Respondents were informed that the participation in the survey was completely voluntary and their data would remain anonymous and would not be linked to personal information, such as their name or address and used solely for scientific purposes. Respondents needed to provide their informed consent at the start of the survey and were required to reconfirm their consent at the end of the survey.

### Questionnaire

The data presented here were part of a larger survey with the primary objective to obtain population tariffs for the CarerQol-7D instrument.

Data were collected on the social–demographic characteristics of all respondents (such as age, gender, education, marital status, and current employment status), the household of the respondent (size, monthly net income), the place of residence (type of residence, region), and the health status of the respondent (self-reported health and EQ-5D-5L). Respondents were asked about their experience with informal care, either as a recipient or a provider (i.e., with response options ‘No’; ‘I have been receiving/have received informal care from a family member or a friend for a longer period of time’; ‘I have been providing/have provided care or support to a family member or a friend for a longer period of time/during the past year/in the past’; ‘I know a person who provides/has provided or receives/received unpaid care’). For the analyses in this paper, we focused on current informal caregivers, i.e., those who were providing informal care at the time of the survey.

Informal caregivers were asked to provide further details on the caregiving situation such as: the nature of health problems of the care recipient (mainly mental, mainly physical, or both); the health status of the care recipient (using the EQ-5D-5L Proxy questionnaire) and whether improvement can be expected in the future; relationship to the care recipient; the weekly time allocated to the caregiving tasks; for how long the respondent had been providing care to the care recipient (duration of caregiving); if the care recipient lived in the same household (if not, the travel time to get there); if the caregiving affected the respondent’s life negatively, positively, or neither negatively nor positively.

### Measurement tools: EQ-5D-5L and CarerQol

The EQ-5D-5L is a generic health status measure that consists of two parts [18]. The descriptive system comprises five dimensions of health (mobility, self-care, usual activities, pain/discomfort, anxiety/depression). Respondents are asked to indicate the problem level (5 levels: 1—no problems, 2—slight problems, 3—moderate problems, 4—severe problems and 5—extreme problems) that best describes their current health status. The digits for the five dimensions can be combined into a 5-digit number that describes the respondent’s health state. Utility scores have been obtained for each health state (EQ-5D-5L index score, value set or tariffs) from representative samples of the general population in several countries, reflecting their preferences for different health states. Due to a lack of country-specific value set for Hungary, we used the UK tariffs in our study (value range: − 0.285 reflecting extreme problems on all dimensions; 1.0 reflecting no problems in any of the five dimensions) [19]. The second part of the questionnaire is the EQ-VAS, which is a vertical a 0–100 visual analogue scale, also called health thermometer. Its two anchors refer to the worst (value 0) and best (value 100) health states that the respondent can imagine. Respondents are asked to value their current health on this scale. The Proxy version of the EQ-5D-5L has been developed for use in situations where patients are not able to report their own health-related quality of life. We applied this version to assess the care recipients’ health status, based on caregivers’ responses. This was done as we did not have access to care recipients.

Informal carers were also asked to complete the CarerQol-7D questionnaire. The CarerQol-7D instrument is a validated tool to measure care-related QoL of informal caregivers [2, 17, 20]. It consists of two separate parts: The CarerQol-7D descriptive system includes two positive (fulfilment and support) and five negative (relational problems, mental health problems, problems combining daily activities with care, financial problems and physical health problems) caregiving dimensions, each with three answering levels (none, some, a lot). Answers to the seven dimensions of the CarerQol-7D, describe the caregiving situation. Utility scores have been obtained for the different CarerQol states from representative samples of the general public in several countries, reflecting their preferences for different states. Utility scores can range from 0 to 100 (representing the worst and the best caregiving situation). In our case, the Dutch population tariffs were used [21] as country-specific value sets are currently not available for the three countries involved in the study. The second part is the CarerQol-VAS, which measures happiness of caregivers on a horizontal visual analogue scale (VAS) ranging from 0 (completely unhappy) to 10 (completely happy).

The Hungarian, Polish and Slovenian language versions of the CarereQol-7D instrument were developed using independent forward–backward translations. The translations were checked for accuracy by native-speaker researchers involved in this study. The survey questionnaire was piloted on five respondents in Hungary.

### Statistical analysis

Descriptive statistics of caregivers and the caregiving situations are calculated and presented in tables and figures. Logistic regression analysis was carried out to further explore the determinants of being an informal caregiver in each country. OLS regressions were carried out to explore the determinants of time spent on informal care per week as well as of the care-related quality of life of caregivers measured by the CarerQol-7D instrument. Control variables included in the regressions were: social–demographic characteristics of caregivers and care recipients, as well as characteristics of caregiving situations.

## Results

### Caregivers and caregiving situations

In each country, of the 1000 respondents (female 51.2, 52.5 and 52.4%; mean age 53.2, 45.1 and 46.4 in Hungary, Poland, and Slovenia, respectively) involved in the survey, a total of 149 (14.9%; 95% CI 12.8–17.3%) in Hungary, 150 (15.0%; 95% CI 12.9–17.4%) in Poland, and 96 (9.6%; 95% CI 7.9–11.6%) in Slovenia reported that they had been providing informal care at the time of the survey. Furthermore, 79/52/42 (HU/PL/SI) respondents reported to had experience with providing informal care prior to the survey, respectively. To allow comparisons with other databases (EQLS, OECD), we also provide the shares of informal caregivers by age groups 18–34, 35–64 and 65+ : 10.3, 14.5 and 18.0% in Hungary, 14.1, 15.9 and 13.8% in Poland and 7.3, 11.5 and 6.0% in Slovenia, respectively. Only considering the subsample of respondents aged 50 and over, the shares of informal caregivers (HU/PL/SI) were 17.0, 16.7 and 11.7%, respectively.

The average age of current informal caregivers was 56.1 years in Hungary, 45.6 in Poland, and 48.0 in Slovenia. The share of women among caregivers was the highest in Hungary (58.4%), while it was 50.0% and 51.0% in Poland and Slovenia, respectively. Additional socio-demographic characteristics of caregivers are presented in Table 1.

**Table 1:** Social–demographic characteristics of informal caregivers

Variables	Hungary		Poland		Slovenia	
	*N*	%	*N*	%	*N*	%
Total	149	100%	150	100%	96	100%
Woman	87	58.4%	75	50.0%	49	51.0%
Age group***						
18–34	14	9.4%	44	29.3%	19	19.8%
35–64	85	57.0%	87	58.0%	68	70.8%
65–	50	33.6%	19	12.7%	9	9.4%
Education***						
Primary	28	18.8%	11	7.3%	11	11.5%
Secondary	55	36.9%	91	60.7%	59	61.5%
Tertiary	66	44.3%	48	32.0%	26	27.1%
Employment***						
Employed full time/self-employed	59	39.6%	64	42.7%	44	45.8%
Working part time	6	4.0%	13	8.7%	6	6.3%
Pensioner	64	43.0%	31	20.7%	21	21.9%
Disability pensioner	5	3.4%	8	5.3%	4	4.2%
Student	1	0.7%	6	4.0%	6	6.3%
Unemployed (seeking for a job)	4	2.7%	7	4.7%	8	8.3%
Unemployed (not seeking for a job)	2	1.3%	5	3.3%	3	3.1%
Homemaker/housewife	3	2.0%	13	8.7%	2	2.1%
Other	5	3.4%	3	2.0%	2	2.1%
Settlement type***						
Capital	32	21.5%	16	10.7%	13	13.5%
Town	85	57.0%	107	71.3%	46	47.9%
Village	32	21.5%	27	18.0%	37	38.5%
Married/in a relationship (yes)	101	67.8%	99	66.0%	72	75.0%
Self-reported health						
Excellent	9	6.0%	8	5.3%	8	8.3%
Very good	28	18.8%	32	21.3%	31	32.3%
Good	67	45.0%	74	49.3%	35	36.5%
Fair	37	24.8%	29	19.3%	16	16.7%
Poor	8	5.4%	7	4.7%	6	6.3%

	Mean	Sd	Mean	Sd	Mean	Sd
Age***	56.1	14.2	45.6	15.5	48.0	14.0
Household size***	2.5	2.5	3.3	3.1	3.0	1.9
Per capita household income EUR***	403	245	362	223	763	542
EQ-5D-5L index***	0.86	0.18	0.77	0.21	0.84	0.19
EQ VAS***	75.0	18.8	65.9	21.9	73.1	21.3

*p < 0.01; **p < 0.05; ***p < 0.1; differences in the distributions across countries were tested by Chi-square test. Differences in means were tested by ANOVA

Characteristics of the caregiving situations and the care recipients are summarised in Table 2. In 78.5%/72.0%/74.0% (HU/PL/SI) of the cases, caregivers had been providing care for more than a year. Caregivers most often provided care to their parents (35.6%/36.7%/41.7%), their parents-in-law (12.1%/8.7%/13.5%), or their partner (12.8%/7.3%/10.4%). Compared to Hungary and Slovenia, in Poland a relatively high share of caregivers provided care to their grandparents (12%). In Hungary and Slovenia, in a majority of the cases, the care recipient did not live in the same household as the caregiver (65.8%/60.4%); while in Poland, 56% did live in the same household. In Hungary, in 49.0% of the cases, the care recipients mainly suffered from physical problems; while in 17.4% of the cases, they mainly suffered from mental problems. A third (33.6%) of the respondents indicated the care recipient suffered from both physical and mental problems, according to the caregivers’ report. These shares were 59.3%/12.7%/28.0% in Poland and 44.8%/15.6%/39.6% in Slovenia.

**Table 2:** Characteristics of caregiving situations

Variables	Hungary		Poland		Slovenia	
	No	%	No	%	No	%
Health problem						
Mostly physical problems	73	49.0%	89	59.3%	43	44.8%
Mostly mental problems	26	17.4%	19	12.7%	15	15.6%
Both physical and mental problems	50	33.6%	42	28.0%	38	39.6%
Health status of the care recipient (reported by the caregiver)***						
Excellent	4	2.7%	2	1.3%	4	4.2%
Very good	4	2.7%	9	6.0%	2	2.1%
Good	25	16.8%	41	27.3%	35	36.5%
Fair	90	60.4%	76	50.7%	31	32.3%
Poor	26	17.4%	22	14.7%	24	25.0%
Health status is***						
Definitive, no improvement is expected in the future (worsening may occur)	108	72.5%	66	44.0%	52	54.2%
Not definitive, it is expected to improve in the future	26	17.4%	43	28.7%	21	21.9%
I do not know	14	9.4%	38	25.3%	16	16.7%
I do not want to answer	1	0.7%	3	2.0%	7	7.3%
Care provided for**						
1 month or less	2	1.3%	4	2.7%	7	7.3%
2–6 months	12	8.1%	20	13.3%	14	14.6%
7–12 months	18	12.1%	18	12.0%	4	4.2%
More than 1 year	117	78.5%	108	72.0%	71	74.0%
Relation of care recipient to caregiver						
Partner	19	12.8%	11	7.3%	10	10.4%
Parent	53	35.6%	55	36.7%	40	41.7%
Child	12	8.1%	13	8.7%	7	7.3%
Other family member	47	31.5%	58	38.7%	34	35.4%
Neighbour/friend	18	12.1%	13	8.7%	5	5.2%
Living in the same household***						
Yes	51	34.2%	84	56.0%	38	39.6%
No	98	65.8%	66	44.0%	58	60.4%
Care experience***						
Rather negative	43	28.9%	23	15.3%	25	26.0%
Neither negative, nor positive	69	46.3%	103	68.7%	60	62.5%
Rather positive	37	24.8%	24	16.0%	11	11.5%

	Mean	Sd	Mean	Sd	Mean	Sd
EQ-5D-5L index care recipient	0.53	0.25	0.49	0.29	0.52	0.32
EQ VAS care recipient**	47.0	22.7	44.4	21.9	48.2	24.2
CarerQol-7D overall score***	76.0	16.2	69.6	17.6	70.9	18.0
CarerQol-VAS	6.8	2.3	6.4	2.1	6.6	2.2
Care time (hours/week)	27.6	35.4	35.5	40.6	28.8	36.9
Travel time for those who live in a separate household (min)	42.1	53.6	48.4	58.2	50.0	61.0

*p < 0.1; **p < 0.05; ***p < 0.01; differences in the distributions across countries were tested by Chi-square test. Differences in means were tested by ANOVA

The average EQ-5D-5L index and EQ VAS scores of the caregivers differed between the three countries: EQ-5D-5L scores of caregivers were 0.86 [95% CI 0.83–0.89] in Hungary, 0.77 [95% CI 0.74–0.81] in Poland and 0.84 [95% CI 0.81–0.88] in Slovenia, while EQ VAS scores were 75.0 [95% CI 72.0–78.1], 65.9 [95% CI 62.3–69.4] and 73.1 [95% CI 68.8–77.4], respectively. In Poland and Slovenia, although the age of caregivers and non-caregivers did not differ significantly, the EQ-5D-5L index and EQ VAS scores of caregivers were significantly lower than those of non-caregivers (mean EQ-5D-5L scores of non-caregivers were: 0.83 SD = 0.19, *F* = 10.17, *p* = 0.0015 in Poland and 0.90 SD = 0.15, *F* = 9.52, *p* = 0.0021 in Slovenia; mean EQ VAS scores of non-caregivers were: 69.1 SD = 21.2, *F* = 2.96, *p* = 0.0857 in Poland and 76.5 SD = 18.0, *F* = 3.00, *p* = 0.0838 in Slovenia). In Hungary, we found no significant differences between caregivers and non-caregivers (mean EQ-5D-5L scores of non-caregivers: 0.87 SD = 0.18, *F* = 0.22, *p* = 0.6413; Mean EQ VAS scores of non-caregivers: 73.7 SD = 20.6, *F* = 0.50, *p* = 0.4779). The average EQ-5D-5L scores of care recipients were 0.53 [95% CI 0.49–0.57] in Hungary, 0.49 [95% CI 0.45–0.54] in Poland and 0.52 [95% CI 0.46–0.58] in Slovenia, while EQ VAS scores were 47.0 [95% CI 43.3–50.7], 44.4 [95% CI 40.9–47.9] and 48.2 [95% CI 43.3–53.1], respectively.

According to the results of the logistic regressions (Table 3, columns 1–3), in Poland and Slovenia, lower EQ-5D-5L scores (worse health status) were significantly associated with the probability of being a caregiver. In addition to this, odds ratios of being a caregiver were significantly higher for women in Hungary and those having tertiary education in Poland. Other variables such as type of residence, age, employment status marital status, household size or income were not significantly associated with caregiver status.

**Table 3:** Results of the regression analyses

Variables	(1)	(2)	(3)	(4)	(5)	(6)	(7)	(8)	(9)
	OR	OR	OR	OLS	OLS	OLS	OLS	OLS	OLS
	Being a caregiver HU	Being a caregiver PL	Being a caregiver SI	Care time HU	Care time PL	Care time SI	CarerQol score HU	CarerQol score PL	CarerQol score SI
Caregivers’ characteristics									
Woman (base: man)	1.689** (1.009–2.828)	0.863 (0.594–1.254)	0.952 (0.578–1.568)	6.822 (1.312)	9.939 (1.557)	17.90** (2.040)	4.366* (1.836)	0.222 (0.0796)	− 0.165 (− 0.0356)
Age	1.012 (0.991–1.032)	1.000 (0.987–1.012)	1.011 (0.991–1.031)	− 0.321 (− 1.282)	− 0.123 (− 0.399)	− 0.244 (− 0.588)	0.0264 (0.199)	0.147 (1.146)	0.347 (1.591)
Education: primary (base: secondary)	1.035 (0.564–1.897)	0.649 (0.306–1.379)	0.545 (0.215–1.383)	2.562 (0.365)	− 13.67 (− 1.062)	0.975 (0.0326)	3.593 (1.197)	− 7.633* (− 1.806)	− 11.29 (− 1.169)
Education: tertiary (base: secondary	1.404 (0.832–2.370)	1.937*** (1.269–2.957)	0.971 (0.556–1.698)	− 0.925 (− 0.117)	− 5.510 (− 0.793)	− 6.970 (− 0.685)	0.402 (0.127)	− 1.988 (− 0.618)	2.023 (0.424)
Working (base: no paid job)	1.049 (0.574–1.917)	0.908 (0.600–1.375)	0.773 (0.440–1.358)	− 13.01* (− 1.975)	4.240 (0.531)	− 20.04*** (− 2.672)	0.349 (0.0945)	0.754 (0.278)	− 2.341 (− 0.492)
Living in a village (base: living in a town)	0.784 (0.430–1.430)	0.904 (0.559–1.464)	1.430 (0.835–2.451)	− 8.329 (− 1.232)	5.260 (0.534)	− 16.19 (− 1.274)	− 3.005 (− 0.920)	− 2.010 (− 0.492)	3.703 (0.711)
Living in the capital (base: living in a town)	0.721 (0.367–1.417)	0.719 (0.378–1.371)	0.863 (0.406–1.836)	11.59 (1.022)	0.785 (0.0826)	− 17.04 (− 1.265)	− 2.786 (− 0.894)	− 1.952 (− 0.293)	− 9.691 (− 1.247)
Married/partner (base: not married)	1.052 (0.624–1.772)	0.874 (0.589–1.297)	1.501 (0.843–2.671)	− 0.0798 (− 0.00857)	12.70* (1.938)	7.947 (0.843)	1.802 (0.542)	0.700 (0.205)	0.0541 (0.0128)
HH size	0.959	1.016	1.023	− 2.615	− 2.776**	− 0.139	2.364*	1.171**	− 0.992
	(0.822–1.120)	(0.969–1.066)	(0.892–1.173)	(− 0.890)	(− 2.347)	(− 0.0383)	(1.938)	(2.117)	(− 0.646)
HH income per capita	1.000 (0.999–1.001)	1.000 (0.999–1.001)	1.000 (0.999–1.000)	− 0.0248 (− 1.648)	− 0.00573 (− 0.316)	− 0.0304** (− 2.232)	0.0121 (1.414)	0.0235*** (2.945)	0.00147 (0.172)
EQ-5D-5L index score	1.128 (0.265–4.811)	0.291*** (0.130–0.655)	0.226** (0.0708–0.721)	16.58 (1.021)	12.83 (0.705)	30.12 (1.188)	44.05*** (5.551)	17.28** (2.141)	34.72** (2.192)
Care recipients’ characteristics									
Mainly mental health problems (base: only physical)				2.242 (0.290)	− 16.67* (− 1.831)	18.65 (1.653)	− 13.78*** (− 3.657)	− 14.67*** (− 3.024)	0.377 (0.0633)
Mental and physical health problems (base: only physical)				2.732 (0.372)	− 1.193 (− 0.113)	16.56* (1.960)	− 7.752** (− 2.597)	− 7.715* (− 1.950)	1.805 (0.341)
EQ-5D-5L Proxy index score of care recipient				− 24.04* (− 1.880)	− 15.44 (− 1.076)	0.419 (0.0201)	8.965 (1.395)	3.465 (0.634)	12.96 (1.217)
Health state is definitive: no (base: definitive)				− 8.444	1.590	− 8.218	7.246*	3.080	− 3.597
				(− 0.910)	(0.173)	(− 0.805)	(1.903)	(0.771)	(− 0.581)
Health state is definitive: do not know (base: definitive)				15.16 (1.046)	− 6.711 (− 0.760)	5.908 (0.483)	− 2.351 (− 0.529)	3.241 (0.860)	− 0.883 (− 0.133)
Caregiving situations’ characteristics									
Duration: since 6–12 months (base: < 6 months)				1.793 (0.177)	2.509 (0.333)	30.36 (1.386)	15.18*** (3.313)	− 0.249 (− 0.0452)	− 5.371 (− 0.438)
Duration: more than 12 months (base: < 6 months)				6.972 (0.762)	20.54** (2.482)	26.35 (1.389)	12.39*** (3.469)	− 0.0394 (− 0.00868)	− 1.532 (− 0.275)
Caregiver’s partner (base: other family member)				4.863 (0.353)	10.75 (0.463)	− 11.29 (− 0.790)	− 2.188 (− 0.429)	− 0.387 (− 0.0611)	− 2.773 (− 0.361)
Caregiver’s parent (base: other family member)				0.287 (0.0377)	− 2.009 (− 0.244)	− 4.909 (− 0.417)	3.451 (1.103)	− 2.939 (− 0.792)	1.307 (0.295)
Caregiver’s child (base: other family member)				− 7.872 (− 0.493)	11.91 (0.901)	13.95 (0.646)	13.49*** (2.964)	− 4.725 (− 0.891)	− 16.23* (− 1.810)
Caregiver’s friend/neighbour (base: other family member)				− 1.997 (− 0.257)	28.49* (1.706)	− 31.57 (− 0.966)	6.526 (1.289)	− 0.994 (− 0.186)	− 3.237 (− 0.297)
Living in the same HH (base: different HH)				34.22*** (2.660)	22.14*** (3.299)	30.95** (2.402)	− 8.936** (− 2.112)	2.173 (0.659)	1.977 (0.281)
Care time (hours/week)							0.0605* (1.909)	0.00250 (0.0610)	0.0469 (0.823)
Constant	0.0363*** (0.00622–0.212)	0.734 (0.212–2.537)	0.266 (0.0359–1.968)	36.48 (1.289)	− 0.0590 (− 0.00253)	− 15.85 (− 0.375)	4.385 (0.252)	38.33*** (3.553)	24.14 (1.217)
Observations	842	914	872	134	144	81	134	144	81
*R*-squared				0.378	0.307	0.447	0.640	0.293	0.388
*F* > test				2.375	2.362	2.652	17.18	2.616	3.672

Columns 1–3: robust 95% confidence intervals are in parentheses; columns: 4–9: robust standard errors are in parentheses. The number of observations in the OLS regression is lower than the number of caregivers in each country due to missing values for the income question

*HH* household

***p < 0.01, **p < 0.05, *p < 0.1

### Time spent on informal care

The average time spent on informal care per week was 27.6 [95% CI 25.1–35.0] (35.5 [95% CI 29.3–40.2]/28.8 [95% CI 23.1–35.6]) hours in Hungary (Poland/Slovenia). In addition to this, for those not living in the same household as the care recipient, the average travelling time to get to the care recipient’s home ranged between 42 and 50 min in the three countries (Table 2).

The results of the OLS regressions showed that, in Hungary and Slovenia, those caregivers who were working (or studying) at the time of the survey provided significantly less hours of care compared to those without a paid job (13 and 20 h less, respectively). In Slovenia, women provided significantly more hours of care than men (i.e., 18 h more on average). In Poland, being married and living in a smaller household were significantly associated with more time spent on informal care. Income was significantly (negatively) associated with care time in Slovenia.

Regarding the caregiving situation, living in the same household was associated with significantly more time spent on informal care in all the three countries (i.e., 34, 22, and 31 h more per week in Hungary, Poland, and Slovenia, respectively). In Hungary, a higher EQ-5D-5L index of the care recipient was significantly associated with less care provided. In Poland, caring for a care recipient for more than 12 months and caring for a neighbour or friend were significantly associated with more time spent on informal care, while providing care for a person with only mental health problems was associated with significantly less hours. In Slovenia, providing care to a person with both physical and mental health problems was associated with significantly more hours spent on informal care.

### Quality of life of caregivers

**Fig. 1:**
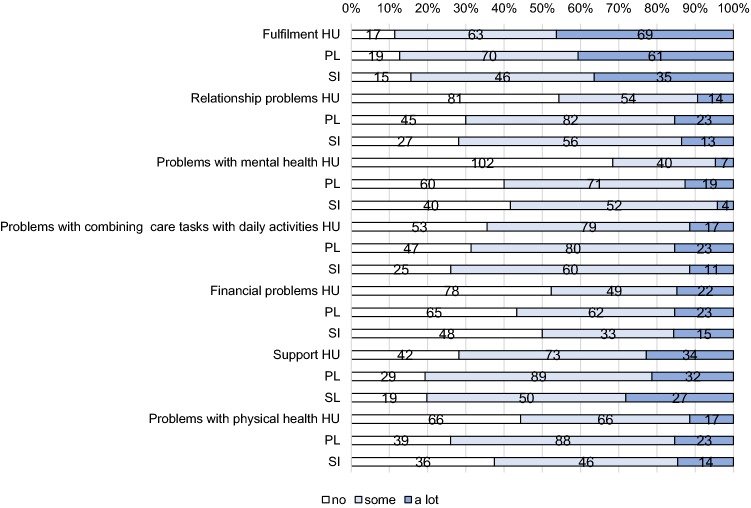
Distribution of answers on the CarerQol-7D dimensions. HU Hungary (*N* = 149), PL Poland (*N* = 150), SI Slovenia (*N* = 96)

The distributions of answers per CarerQol-7D domain are presented in Fig. 1. In Hungary, most problems were reported in the domains “combining care tasks with daily activities” (64% of respondents reported some or a lot of problems) and “physical health” (56%). In Poland, the most problems were reported in the domains “physical health” (74%) and “relationship with the care recipient” (70%). In Slovenia, most problems were reported in the domains “problems with combining care tasks with daily activities” (74%) and “relationship with the care recipient” (72%). Least problems were reported with own mental health (32%) in Hungary, and with finances in Poland (57%) and Slovenia (50%).

Regarding the positive CarerQol domains, 89% (87%, 84%) of caregivers experienced some or a lot of fulfilment in Hungary (Poland, Slovenia); while, respectively, 72, 81 and 80% reported to receive some or a lot of support if needed.

As shown in Table 2, the CarerQol-7D utility scores differed substantially across the three countries (76.0 [95% CI 73.3–78.6] in Hungary, 69.6 [95% CI 66.7–72.4] in Poland and 70.9 [95% CI 67.3–74.5] in Slovenia). The CarerQol-VAS happiness scores were more similar (with 6.8 [95% CI 6.4–7.2], 6.4 [95% CI 6.0–6.7] and 6.6 [95% CI 6.1–7.0], respectively).

According to the regression results (in Table 3), higher EQ-5D-5L scores (better health) of caregivers were associated with higher CarerQol-7D scores in all the three countries. Living in a larger household was significantly associated with higher CarerQol-7D scores in Hungary and Poland. In Hungary, women reported significantly higher CarerQol-7D scores than men. In Poland, primary education and lower per capita household income were associated with significantly lower CarerQol-7D scores.

Regarding caregiving situations, caring for a patient with mental health problems (considering both subgroups of ‘mainly mental’ and ‘physical and mental’ problems) was associated with significantly lower CarerQol-7D scores in Hungary and Poland. In Hungary, caring for a person for more than 6 months, living in a separate household than the care recipient, potential improvement of the health of the care recipient, and hours spent on informal care were all significantly and positively associated with CarerQol-7D scores. Regarding the relationship with the care recipient, caring for a child of any age (compared to other family members) was associated with a significantly higher CarerQol-7D score in Hungary, but lower scores in Slovenia.

## Discussion

In this study, we provided comparative descriptions of informal care and explored the determinants of (the impact of) being an informal caregiver in three CEE countries, namely Hungary, Slovenia and Poland. Associations between caregivers’ characteristics, caregiving situations and the time spent on informal care were investigated. This was the first study using the CarerQol-7D instrument to measure the care-related QoL of caregivers in Hungary, Poland and Slovenia among the general population. Another novelty of this research was that the health status of both caregivers and care recipients was assessed with the EQ-5D-5L questionnaire.

### Informal caregivers

We found that in our online sample, 14.9% of the adult population had been providing informal care to a relative or a friend in Hungary, while this share was 15% in Poland and 9.6% in Slovenia. These percentages are lower than those reported by the interview-based EQLS among the population aged 18 and over (18, 20 and 15%, respectively) [15]. For the subgroups of respondents aged 50 and over, the proportions of caregivers were close to those reported by the OECD for the same age group in Hungary (17.0% vs. 16.2%), somewhat higher than those reported in Poland (16.7% vs. 12.8%), and slightly lower than those reported for Slovenia (11.7% vs. 14.6%). For Hungary, Rubovszky in 2017 [16] provided a much higher estimate in a representative, phone interview-based study (25.5%).

According to our results, informal caregivers have diverse socio-demographic backgrounds, both within and across countries. However, some typical features deserve mentioning. Informal care providers most often were mostly in the age category 35–64 (i.e., working age), and provided care for their parents (in-law). Most care recipients suffered from physical problems in all the three countries; however, a substantial percentage (28–40%) suffered from both physical and mental health problems. Care recipients’ EQ-5D-5L scores varied between 0.49 and 0.53, and the EQ VAS score between 44.4 and 48.2 across the countries. This highlights their substantial health problems. Due to lack of population norms in Hungary and Slovenia, we could not compare the health status of the caregivers to that of the general public. The average EQ VAS score of the caregivers was slightly lower in Hungary, nearly identical in Slovenia and slightly higher in Poland than that of the respective age group in the general population [22]. In Poland and Slovenia, the EQ-5D-5L scores and EQ VAS scores of caregivers were significantly lower than those of non-caregivers in our sample. No significant difference between caregivers and non-caregivers was observed in Hungary.

Results of regression analyses revealed that Hungarian women had a higher probability of being a caregiver. Having tertiary education was associated with a higher probability of being a caregiver in Poland. EQ-5D-5L scores of caregivers were (negatively) associated with caregiver status in Poland and Slovenia. Other variables such as type of residence, age, employment status, marital status, household size, or income were not significantly associated with caregiver status. Previous studies report that women are more likely to be an informal caregiver, for example, OECD Health at Glance reports that the share of women among informal caregivers (among the population 50+), is 71.0% in Hungary, 64.6% in Poland 60.6% in Slovenia [14]. In contrast, the EQLS survey reported similar rates for males and females in Hungary [15]. For Hungary, Rubovszky reported that informal caregiver tend to be middle-aged women with children, living in deprived areas with a below-average household income [16]. However, in our samples, age and type of residence were not significantly associated with being a caregiver.

### Time spent on informal care

In our study, we found caregivers to spend an average of 27.6 h per week on informal care in Hungary, 35.5 in Poland, and 28.8 in Slovenia. This corresponds to 69, 89 and 72% of a usual 40-h workweek (in a paid job). As a large majority of caregivers were working or studying, these findings suggest that care hours substantially reduced leisure time. Moreover, a majority of caregivers did not live together with the care recipient in Hungary and Poland. Hence, they had to travel regularly to provide care, which took substantial amounts of time as well. In about two-thirds of the cases, care lasted for more than 1 year, indicating a longstanding strain on involved caregivers. For Hungary, Rubovsky [16] reported an average of 26.7 h per week spent on care, which closely resembles our findings. For further comparison, Beretzky and Péntek [6] reported average informal care time (hours/week) in Hungary for several chronic conditions. Among those who reported to receive informal care, the highest number of informal care hours was reported for caring for dementia and epilepsy patients (82.8 and 40.9 h/week), followed by patients with benign prostatic hyperplasia (23.2 h/week) and multiple sclerosis (20.5 h/week). Informal care time varied between 15 and 20 h per week in systemic sclerosis, psoriatic arthritis, and Parkinson-disease and between 10 and 15 h per week for psoriasis, rheumatoid arthritis, endometriosis, schizophrenia, AMD and osteoporosis.

Living in the same household as the care recipient was associated with significantly more time spent on informal care in all the three countries. Caregivers with paid jobs provided less hours of care in Hungary and Slovenia. Other variables were not systematically associated with time spent on caregiving in the three countries. Only in Hungary, informal care time was found to increase with worse health status of the care recipients, as measured with the EQ-5D-5L instrument. A similar association was found by Beretzky and Péntek [6]. The Hungarian results, moreover, are in line with an algorithm to estimate the amount of informal care hours based on EQ-5D data [23].

### Quality of life of informal caregivers

The mean CarerQol-7D scores were 76.0 in Hungary, 69.6 in Poland and 70.9 in Slovenia. The CarerQol-VAS happiness scores were 6.8, 6.4 and 6.6, respectively. Happiness scores are similar to the average happiness scores of the total population measured by the EQLS 2016 in Hungary and Slovenia (7 and 7) but slightly lower in Poland (8) (considering methodological differences, as happiness was measured on a 1–10 scale in the EQLS survey, while on a 0–10 scale in our study by the CarerQol-VAS instrument) [24]. For further comparison, the CarerQol-7D scores and CarerQol-VAS happiness scores of caregivers of Alzheimer patients from eight European countries were reported in the same range (between 72–80; and 5.6–7.0 across countries) [25]. Higher scores were reported in a multi-centre study with caregivers of children with drug-resistant epilepsy (81.4/6.9; *N* = 181) [26] and among caregivers of Dutch elderly after a hip fracture (83.7/7.6, *N* = 123) [27]. On the other hand, somewhat lower scores were reported for caregivers providing palliative care in Australia (73.5/5.8; *N* = 97) [28].

The care-related QoL of caregivers (CarerQol-7D) was significantly associated with the health status of the care recipient in all countries, as was also found in previous studies [26, 29]. Furthermore, in Hungary and Poland, we observed lower care-related QoL for those who were caring for patients with mental health problems. Similarly, Karg et al. reported that caring for a relative with dementia was associated with poorer health and lower QoL than caring for non-dementia patients [30]. Furthermore, in Hungary, caring for someone longer than 6 months was associated with higher care-related QoL of caregivers, which might indicate adaptation to the caregiving situation or relate to underlying care needs. Also, time spent on caring and whether the caregiver and the care recipient lived in the same household were associated with CarerQoL scores in Hungary.

## Limitations

Regarding the limitations of our study, some issues deserve noting. First, although we aimed to include representative samples, at least for the internet using population in the three countries, some population groups were over- and underrepresented in our final samples. This may also be the case for (specific) informal caregivers, since one may expect (burdened) caregivers to be less likely to voluntarily participate in an online survey. Second, performing a web-based survey may have further influenced our results in terms of how engaged respondents were in answering the survey. Third, we did not distinguish between primary and other caregivers. Hence, if a person indicated to provide care for someone, and identified themselves as informal caregivers, other caregivers could also be involved in caring for the same care recipient (and potentially have an even higher burden of caregiving). Focusing only on primary caregivers (as is often the case in clinical contexts) may lead to different results. Fourth, even though our total samples of 1000 per country were sizeable, sample sizes of the subgroups of caregivers were relatively small, which limits the statistical power of our analyses, also in relation to the large number of variables included in the regressions [31]. The relatively small sample sizes may also explain some of the differences observed between countries. We have carried out robustness checks applying step-wise approach for the OLS regressions and also a combined analysis for the three countries, both providing similar results (tables are available on request from the authors). Fifth, we obtained information on the care recipient only through the caregivers, which may have resulted in less accurate descriptions of their health and well-being. Sixth, we used translated versions of the CarerQol instrument and other survey questions. Further in-depth research is needed on the construct validity of these translations. Moreover, we used the Dutch valuation set for the CarerQol and UK tariffs for the EQ-5D-5L instrument. This may have impacted results and future research should focus on the development of population tariffs for both instruments in CEE countries. These would provide more accurate reflections of preferences in the respective countries. Finally, while already elaborate, our study focused on some elements of the burden of informal care, leaving out other elements like direct costs of caregivers (e.g., for travel or home adaptations), reduced labour force participation, as well as information on the (supplementary or complementary) utilisation of formal long-term care and other health care services by care recipients.

## Concluding

In summary, we confirmed that informal caregiving is a common phenomenon in the CEE region. The amount of time spent on informal care is substantial, often in combination with paid work, underlining the substantial burden on caregivers. This can lead to important well-being and health effects. Indeed, in Poland and Slovenia, we observed significantly lower health scores in caregivers compared to non-caregivers. This emphasises the need for more research and knowledge in this area, also given the reliance of many health care systems on informal caregivers in the total provision of care.

This was the first study using the CarerQol-7D instrument to measure care-related quality of life of informal caregivers in the general population in Central-Eastern Europe. The CarerQol-7D appears to be a valid instrument to measure care-related QoL in the general population, showing a strong association with the EQ-5D-5L instrument. Our results suggest that caring for people with mental health problems may impact care-related QoL negatively. Hence, more attention and support could be provided to those caregivers, for instance by implementing targeted mental health policies and training and supporting caregivers in this context. More attention paid to informal care in research, policy and clinical practice remains warranted. The fact that this care is often unpaid does not mean it is free. It can come at the high price of sacrificing resources, time, well-being and health and is of great value to patients and society.
